# Isolated oligohydramnios in previous pregnancy is a risk factor for a placental related disorder in subsequent delivery

**DOI:** 10.1186/s12884-022-05230-9

**Published:** 2022-12-06

**Authors:** Sophia Leytes, Michal Kovo, Eran Weiner, Hadas Ganer Herman

**Affiliations:** 1 Department of Obstetrics & Gynecology, the Edith Wolfson Medical Center, Holon, Israel; 2 Department of Obstetrics & Gynecology, the Meir Medical Center, Kfar Saba, Israel

**Keywords:** Oligohydramnios, Fetal growth retardation (FGR), Small for gestational age (SGA), Placenta

## Abstract

**Background:**

We aimed to assess the association between isolated oligohydramnios in previous pregnancy and the incidence of placental related complications in subsequent pregnancy.

**Methods:**

This was a retrospective cohort study of live singleton births from a single university affiliated medical center during an eleven-year period of women with two subsequent deliveries at our center. An analysis of outcomes was performed for all second deliveries, comparing women for whom their first delivery was complicated by isolated oligohydramnios (previous oligohydramnios group), and women without isolated oligohydramnios in their first delivery (control group). Patients for whom their first delivery was complicated by small for gestational age, pregnancy induced hypertension and preterm birth were excluded. The study groups were compared for obstetric and early neonatal outcomes, recurrence of oligohydramnios and a composite of placental related pregnancy complications.

**Results:**

A total of 213 in the previous oligohydramnios group and 5348 in the control group were compared. No differences were found between the groups in maternal age, body mass index, smoking and comorbidities. Gestational age at delivery was, 39.6 ± 1.3 vs. 39.3 ± 1.4 weeks, *p* = 0.006, in the previous oligohydramnios and controls respectively, although preterm birth rate was similar between the groups. The previous oligohydramnios group had a significantly higher incidence of oligohydramnios in second delivery, aOR 3.37, 95%CI 1.89–6.00, small for gestational age neonates, aOR 1.94, 95% CI 1.16–3.25, and overall placental related disorders of pregnancy, aOR 2.13, 95%CI 1.35–3.35.

**Conclusion:**

Pregnancies complicated by isolated oligohydramnios are associated with an increased risk of placental related disorders in subsequent pregnancy. Isolated oligohydramnios may be the first sign of placental insufficiency and an independent manifestation of the placental related complications spectrum.

## Background

Amniotic fluid amount is an important parameter for the evaluation of fetal well-being. There is no consensus regarding the best method to estimate the amount of amniotic fluid [1], and the definition of oligohydramnios. Some define oligohydramnios by a deepest vertical pocket (DVP) of less than 2 cm, while others prefer calculating a four-quadrant amniotic fluid index (AFI) and define oligohydramnios as a value of 5 cm or less [1].

Amniotic fluid volume is dynamic. Its production increases gradually until 32 weeks of pregnancy and remains stable until term. The main sources of amniotic fluid are fetal urination and lung secretions [2]. Oligohydramnios most commonly occurs secondary to premature rupture of membranes. Yet, it has also been related to post-term pregnancies, intrauterine fetal growth retardation (FGR), maternal chronic illness, fetal malformations and chromosomal anomalies. Finally, oligohydramnios may be isolated. Overall incidence of isolated oligohydramnios is between 0.5 and 5%, and depends on local definitions and target population [3–5]. Isolated oligohydramnios is a diagnosis of exclusion, after comprehensive assessment of the fetus to exclude malformations, genetic anomalies and FGR. Moreover, a detailed and thorough maternal examination is necessary to rule out premature rupture of membranes, intrauterine infection or chronic maternal illness. Often oligohydramnios itself serves as an indication for induction of labor [6] and entails an increased risk of operative delivery and cesarean section for fetal distress [7]. Other studies have not demonstrated an increased risk of obstetric interventions or immediate neonatal outcome impairment [5].

The pathogenesis of isolated oligohydramnios is unclear, yet it has been viewed by some as an expression of placental insufficiency, and as such is regarded as an additional placental related obstetrical syndrome, in similar to FGR and preeclampsia [8]. Others view this condition as a benign finding, necessitating intervention only in the presence of additional findings. It is well established that placental syndromes such FGR and preeclampsia tend to recur and increase the overall risk of placental complications in subsequent pregnancy [9, 10]. We therefore set to examine if isolated oligohydramnios displays similar recurrence risks as other placental mediated complications and may be regarded as risk factor for subsequent pregnancies. Our objectives were to evaluate the correlation between isolated oligohydramnios in previous pregnancy and its recurrence in subsequent pregnancy, and the incidence of placental-related complications in subsequent pregnancy.

## Methods

This was a historic cohort at a single university-affiliated center – the Edith Wolfson Medical Center between November 2008 and December 2019. The center serves a population of half a million citizens from the area, and, in similar to alternative center in the area, offers a maternal fetal division and neonatal intensive care if needed. Women with a first and second livebirth singleton delivery at our center during these years were included in the cohort. All singleton pregnancies with a diagnosis of isolated oligohydramnios were identified from electronic medical records and cases reviewed to exclude any secondary causes for oligohydramnios, including premature rupture of membranes, intrauterine fetal growth retardation (FGR), maternal chronic illness, fetal malformations and chromosomal anomalies. We excluded women with any delivery complicated by fetal chromosomal or genetic abnormalities, by birth before 24 weeks, women with lack of prenatal care and cases of current or past intrauterine fetal death. We also excluded women whose first delivery was complicated by small for gestational age, preeclampsia, preterm birth or placental abruption, all placental related complications that are known to recure in subsequent pregnancies [9–12].

We compared pregnancy and neonatal outcomes of two groups – second deliveries of women with isolated oligohydramnios in their first delivery (previous oligohydramnios group), and second deliveries of women without isolated oligohydramnios in their first delivery (control group).

As per institutional protocol, all women admitted to our obstetric emergency room and delivery ward underwent assessment of amniotic fluid amount by ultrasound as an integral part of obstetric evaluation. Amniotic fluid was assessed by obstetricians, with the use of abdominal probes (Voluson E8, GE Healthcare, Milwaukee, WI). Oligohydramnios was determined in the presence of a DVP of less than two centimeters or an AFI of less than 5 cm, and coding was entered to the computerized system as per the ICD9 (code 658.03).

Subsequently, evaluation was undertaken as permitted for premature rupture of membranes, fetal growth, and fetal malformations/genetic abnormalities. FGR was determined in the presence of an estimated fetal weight of less than the 10th percentile using local population-based nomograms [13]. In accordance with national guidelines, induction of labor in cases of isolated oligohydramnios is indicated at 41 + 0 weeks of pregnancy provided reassuring fetal and maternal status until then [14], although for women with a favorable Bishop score, induction of labor was offered as of 39 + 0 weeks. If expectant management was chosen, surveillance with biophysical testing and a non-stress test were undertaken twice a week until delivery. Mode of delivery was dictated by routine obstetrical indications. At our institution for all uncomplicated pregnancies induction of labor is offered at 41 weeks.

Gestational age was determined as based on last menstrual period and adjusted in case of a 5–7-day discrepancy from first trimester ultrasound assessment. Small for gestational age (SGA) neonates were defined as a neonatal birthweight of less than 10th percentile according to local normograms [13]. Pregnancy induced hypertension included gestational hypertension and preeclampsia. Gestational hypertension was diagnosed with an elevated systolic blood pressure 140 mmHg or more or a diastolic blood pressure of 90 mmHg or more, or both, on two occasions at least 4 hours apart after 20 weeks of gestation in a woman with a previously normal blood pressure [15]. Preeclampsia was defined with new onset hypertension with measurements as mentioned previously and proteinuria or an end-organ dysfunction with or without proteinuria after 20 weeks of gestation [15]. Preterm birth was defined as a delivery which occurred prior to 37 weeks gestation. Diagnosis of placental abruption was made based on clinical presentation and confirmed postpartum by placental examination. We defined a composite of any placental complication, defined as one of – pregnancy induced hypertension, small for gestational age, preterm delivery and placental abruption.

### Data collection

Data from the subsequent pregnancy and delivery were collected from patients’ computerized medical records. The following demographic characteristics were obtained: maternal age, gravidity, parity, body mass index (BMI kg/m^2^), assisted reproductive technique use, smoking, pregestational diabetes mellitus (DM), chronic hypertension and rate of uterine Mullerian anomalies. The following obstetric outcomes were assessed: gestational age at delivery, mode of delivery, preeclampsia, gestational hypertension and DM, placental abruption, recurrence of oligohydramnios and intrapartum or postpartum need for maternal blood transfusion. The following data were collected from the neonatal records: birthweight and Apgar scores at 5 mins.

### Statistical analysis

Data were analyzed with Epi Info, version 7.0 (Centers for Disease Control and Prevention, Atlanta, GA). Continuous variables were calculated as mean ± standard deviation or median and range or interquartile range (IQR) as appropriate and compared with the use of the Student t-test or Mann-Whitney test. Categorical variables were calculated as number (%) and compared using the Chi-square test or Fisher’s exact test as appropriate. All tests were two sided, and *p* value of less than 0.05 was considered statistically significant. Logistic regression analyses were employed to control for confounders as indicated.

### Power calculation

The primary outcome was defined as a composite of the main placental-related complications – SGA, preeclampsia, preterm birth or placental abruption. We assumed a 5% incidence of isolated oligohydramnios as previously reported [3, 4], and accordingly a 1:19 ratio between women with and without oligohydramnios in their first delivery. Thus, to demonstrate a 20% incidence of placental complications following oligohydramnios in previous pregnancy, as compared to an assumed 10% without oligohydramnios, a minimal sample size of 92 women with oligohydramnios in primary pregnancy and 1740 without were calculated to suffice, with an 80% power an 0.05 alpha. The number of cases available for inclusion eventually proved larger than the minimal number needed.

Our study was approved by the Edith Wolfson institutional Ethics Committee on April 1st, 2021, institutional review board approval number 0013–21-WOMC. All methods were carried out in accordance with relevant guidelines and regulations.

## Results

During the study period 5561 second deliveries occurred at our institution and were eligible for inclusion - 213 deliveries with isolated oligohydramnios in previous delivery (3.8% of all deliveries) and 5348 pregnancies in the control group, with no oligohydramnios in previous delivery.

Demographic characteristics of the groups are presented in Table 1. There were no differences in maternal age, BMI (kg/m^2^), rate of smoking and comorbidities between the groups.

**Table 1:** Demographic characteristics of the oligohydramnios and control groups

	Previous oligohydramnios *n* = 213	Control group *n* = 5348	*P*
Age, (years), mean ± SD	28.6 ± 4.4	28.1 ± 4.4	0.15
BMI (kg/m^2^), mean ± SD	23.7 ± 4.7	23.6 ± 4.7	0.77
Gravidity, median (range)	2 (2–6)	2 (2–11)	0.51
In vitro fertilization, *n* (%)	5 (2.3%)	174 (3.2%)	0.46
Smoking, *n* (%)	34 (15.9%)	706 (13.2%)	0.24
Pre gestational DM, *n* (%)	1 (0.4%)	25 (0.4%)	> 0.99
Chronic hypertension, *n* (%)	1 (0.4%)	11 (0.2%)	0.37
Mullerian abnormality, *n* (%)	1 (0.4%)	18 (0.3%)	0.52

*SD* standard deviation, *n* Number, *BMI* body mass index, pre-gestational, *DM* diabetes mellitus

Obstetric and neonatal outcomes of the previous oligohydramnios and the control groups are presented in Table 2. Deliveries in the previous oligohydramnios group were notable for a higher gestational age, 39.6 ± 1.3 vs. 39.3 ± 1.4 weeks, *p* = 0.006, yet preterm birth rate was similar between the groups. After adjustment for gestational age, the previous oligohydramnios group had a significantly higher rate of oligohydramnios, 6.5% vs. 1.9%, aOR 3.37, 95%CI 1.89–6.00, composite placental disorders, 12.2% vs. 8.5%, aOR 2.13, 95%CI 1.35–3.35 and small for gestational age neonates, 7.9% vs. 4.2%, unadjusted OR OR 1.94, 95% CI 1.16–3.25.

**Table 2:** Obstetric and neonatal outcome of the oligohydramnios and control deliveries

	Previous oligohydramnios *n* = 213	Control group *n* = 5348	*P*	aOR
Gestational age, (weeks), mean ± SD	39.6 ± 1.3	39.3 ± 1.4	0.006	
Premature delivery, *n* (%)	4 (1.8%)	172 (3.2%)	0.42	
Pregnancy induced hypertension, *n* (%)	4 (1.8%)	45 (0.8%)	0.11	2.56 (0.90–7.24)
Oligohydramnios in current pregnancy, *n* (%)	14 (6.5%)	106 (1.9%)	< **0.001**	3.37 (1.89–6.00)
Gestational diabetes mellitus, *n* (%)	7 (3.2%)	163 (3.0%)	0.84	1.17 (0.54–2.54)
Placenta previa, *n* (%)	1 (0.4%)	13 (0.2%)	0.42	2.71 (0.34–21.30)
Placental abruption, *n* (%)	1 (0.4%)	37 (0.6%)	> 0.99	0.86 (0.11–6.37)
Instrumental delivery, *n* (%)	6 (2.8%)	119 (2.2%)	0.56	1.22 (0.53–2.82)
Cesarean delivery, *n* (%)	43 (20.1%)	830 (15.5%)	0.06	0.96 (0.60–1.54)**
Blood transfusion ante / postpartum, *n* (%)	2 (0.9%)	39 (0.7%)	0.67	1.30 (0.31–5.45)
Birth weight, (grams), means ± SD	3301 ± 376	3324 ± 435	0.25	
Small for gestational age, *n* (%)	17 (7.9%)	228 (4.2%)	**0.009**	
Five-minute Apgar score < 7, *n* (%)	0	8 (0.1%)	> 0.99	
Composite placental disorder, *n* (%)	26 (12.2%)	455 (8.5%)	**0.05**	**2.13 (1.35–3.35)**

*SD* standard deviation, *n* number, *aOR* adjusted odds ratio, adjusted for gestational age

Premature delivery – under 37 weeks; Pregnancy induced hypertension – gestational hypertension or preeclampsia

Composite placental disorder included at least one of the following complications: pregnancy induced hypertension, small for gestational age, placental abruption and preterm birth

**Adjusted for gestational age, previous cesarean delivery and composite placental disorder

In an additional regression analysis, hereby described and not featured as a separate table, oligohydramnios in second delivery served as a dependent factor, while oligohydramnios in first delivery, gestational age and placental complication composite served as independent factors. Oligohydramnios in first pregnancy was found independently associated with incidence in second delivery, aOR 3.07, 95%CI 1.71–5.51, *p* < 0.001, as were gestational age, aOR 1.23, 95%CI 1.07–1.43, and placental complications of pregnancy, aOR 3.30, 95%CI 2.02–5.38.

## Discussion

The objective of our study was to evaluate the correlation between isolated oligohydramnios in previous delivery and adverse placental mediated complications in subsequent delivery. We therefore investigated a cohort of second deliveries at our institution as described, excluding previous deliveries with adverse placental mediated outcomes to avoid a confounding effect for subsequent delivery. We demonstrated a significantly higher rate of oligohydramnios, small for gestational age neonates and overall placental disorders in subsequent delivery, following isolated oligohydramnios in previous delivery, as the odds ratio was almost double than of controls. No additional differences were noted in other adverse outcomes.

A debate regarding the pathogenesis and significance of oligohydramnios exists. While some consider this to be a variant of common clinical presentation, especially in the context of term pregnancy, others regard this entity as abnormal, and view it as a form of placental insufficiency. In some studies, isolated oligohydramnios at term was found to be related to higher rates of adverse outcomes like meconium aspiration syndrome, cesarean delivery for fetal distress and admission to NICU [6, 16]. Yet, the interpretation of these results must also account for the effects of iatrogenic interventions and lower weight fetuses. In a different study, expectant management of isolated oligohydramnios at preterm was associated with similar neonatal outcomes as compared to pregnancies with normal AFI, although an increased risk of new onset fetal growth restriction and lower birthweight was noted [17]. This finding may suggest that in women with isolated oligohydramnios, reduced amniotic fluid volume can be the first sign of placental insufficiency. Nevertheless, others did not demonstrate any adverse outcomes with isolated oligohydramnios [5, 7]. An important adverse outcome that could not be evaluated in most studies regarding oligohydramnios was the risk of intrauterine fetal death, due to its rarity and insufficient sample size to address this outcome. Casey et al. showed a significant association between stillbirth and oligohydramnios, that persisted after exclusion of malformed fetuses from the analysis. Yet, they couldn’t confirm that intervention may reduce this risk [18]. Unfortunately, it is unlikely that a randomized interventional trial will ever be able to address this specific complication.

Examination of placental histology often sheds light in the investigation of pregnancy complications, as placental mediated complications are often associated with typical and suggestive findings, such as vascular malperfusion and inflammatory lesions [19]. To investigate both clinical and placental histological aspects of isolated oligohydramnios, Miremberg et al. examined placentas from term pregnancies with isolated oligohydramnios as compared to matched controls [20]. Authors noted an increased rate of low placental weight with isolated oligohydramnios, as was a higher rate of abnormal cord insertions and maternal vascular malperfusion lesions. Neonates in the isolated oligohydramnios group had a lower birthweight and higher incidence of adverse outcomes [20]. These findings support isolated oligohydramnios’ relation to the “placental insufficiency” spectrum and are in line with our findings. An increased risk for small for gestational age neonate in subsequent pregnancy supports the assumption that isolated oligohydramnios is a placental mediated disorder, as it displays a similar risk for placental complications in subsequent pregnancies, as do other hallmark placental disorders.

Our study is limited by its retrospective design, although data did originate from a single center and were available for review. Importantly, information was available for actual birthweight hence SGA incidence was evaluated and not intrauterine growth retardation, hence SGA neonates may include those constitutionally small. In a prospective setting the two would be better differentiated, and neonatal gender, unavailable for the current analysis, would be adjusted for. Additional limitation is the lack of placental pathology supporting placental-related origin of mentioned pregnancy complications.

To strengthen the statistical significance, we had a preliminary power analysis and a relatively large sample size that was mandatory to support our theory. Still, the rate of preeclampsia in our cohort was relatively low, therefore, our study was unable to demonstrate an association between isolated oligohydramnios in first pregnancy and preeclampsia in subsequent delivery if one exists. A distinctive feature of our research is the ability to evaluate the predominant effect of isolated oligohydramnios on future delivery, as we selected a cohort of women without any other obstetric placental-related complication in their previous delivery. Our findings may be useful in the clinical setting indicating the importance of reviewing patients’ obstetrical history. In cases of isolated oligohydramnios in previous delivery, a clinician should consider closer observation of fetal growth in subsequent pregnancy to diagnose FGR in time.

## Conclusions

In conclusion, the current study points to a strong association between isolated oligohydramnios in previous delivery and any main placental disorder in the subsequent delivery, especially oligohydramnios recurrence and small for gestational age neonate. Based on our results the risk for a small for gestational age neonate is increased two-fold in subsequent delivery. Accordingly, isolated oligohydramnios may be the first sign of placental insufficiency even in the absence of overt fetal growth impairment and may be an independent manifestation of the placental related complications spectrum.

Randomized controlled trials with larger sample sizes are needed to confirm our hypothesis, and to define the proper management in cases of isolated oligohydramnios, including establishing guidelines for follow up in subsequent pregnancies.

## Data Availability

The datasets generated and/or analysed during the current study are not publicly available due to institutional regulations, but are available from the corresponding author on reasonable request.

## Abbreviations

AFI: Amniotic fluid index
DVP: Deepest vertical pocket
FGR: Fetal growth retardation
NICU: Neonatal intensive care unit
BMI: Body mass index
DM: Diabetes mellitus
SGA: Small for gestational age
SD: Standard deviation
